# How does green human resource management foster employees’ environmental commitment: A sequential mediation analysis

**DOI:** 10.1016/j.heliyon.2024.e33202

**Published:** 2024-06-17

**Authors:** Saeed Turki Alshahrani, Kamran Iqbal

**Affiliations:** a Imam Mohammad Ibn Saud Islamic University (IMSIU), Saudi Arabia; b Capital University of Science and Technology, Islamabad, Pakistan

**Keywords:** Green HRM, Environmental commitment, Organizational pride, Organizational identification

## Abstract

Drawing on the social identity theory, this study aims to examine the impact of organizational pride and organizational identification as sequential mediators in the association between green HRM and employee environmental commitment. The study extended prior research by incorporating the sequential mediators of organizational pride and organizational identification. The researchers gathered data from 267 employees of telecommunications companies in Pakistan. They used Smart PLS software version 3.0 to carry out partial least squares structural equation modeling to verify the hypotheses. The results indicate that green HRM leads to organizational pride, which, in turn, leads to increased organizational identification and, consequently, enhances environmental commitment. The findings hold significant value to practitioners and HR managers striving to develop HR practices that support sustainability, contribute to a culture of environmental responsibility, and lead to positive green outcomes for employees.

## 1 Introduction

Today's world is facing significant global challenges such as environmental degradation and the exhaustion of natural resources. In response to global pressure, companies worldwide are adopting environmentally friendly business strategies to achieve their strategic objectives. Their active participation in CSR activities not only helps meet the expectations of major stakeholders but also facilitates improved performance outcomes and the acquisition of a competitive advantage in the market. Studies consistently show that Corporate Social Responsibility (CSR) is important for all stakeholders of an organization, including consumers, shareholders, employees, investors, and management [1]. CSR can also provide a competitive advantage for an organization by enhancing its reputation and helping to retain skilled employees [2,3].

Green HRM practices have become one of the tools that modern businesses use to respond to environmental issues [4]. Green HRM and CSR are often closely linked concepts, as both involve considering the impact of a company's operations on the environment and society. Previous research has also identified a strong relationship between human resource management (HRM) and CSR [5]. Kundu and Gahlawat [6] have also emphasized the necessity for organizations to shift their focus from solely prioritizing economic growth to adopting responsible and sustainable human resource management practices. By incorporating sustainability into its HR policies and practices in the form of green HRM, a company can not only improve its environmental performance but also by Implementing such practices can have a range of benefits for organizations. These benefits include improved environmental performance [7], employee satisfaction [8], perceived organizational support [9], job performance [10], in-role and extra-role green behaviors [11] and potentially improved financial performance [12]. In fact, companies introduce green HRM practices to positively influence environmental outcomes [13]. This study will add to the literature on green HRM and sustainability. Moreover, it will help to extend our understanding of the psychological mechanisms that connect green HRM and environmental commitment. The adoption of green HRM practices appears to be an effective way to promote environmental commitment among employees. By aligning HR practices with environmental goals and values, organizations can foster a culture of sustainability and encourage employees to actively support and promote eco-friendly initiatives. Employee environmental commitment is crucial for the success of any organization aiming to implement sustainable practices. When employees are dedicated to reducing their environmental impact and promoting eco-friendly behavior, the company as a whole is more likely to reach its sustainability goals. Additionally, employee commitment to environmental issues can lead to increased innovation and efficiency, as well as improved employee morale and engagement.

While extensive research has established a strong relationship between green HRM and environmental commitment [14,15] there is a gap in the literature regarding the process through which green HRM influences employees' environmental commitment, as the process is rarely explored in the literature. This study aims to address this gap by exploring the mechanism that connects and clarifies the relationship between green HRM and environmental commitment. The primary aim of this research is to inspect the process through which employees’ understanding of green HRM practices influences their environmental commitment as a result of feeling proud of their organizational membership, which, in turn, enhances their organizational identification and leads to a higher environmental commitment. The research question that will guide this study is: *What are the underlying mechanisms and processes through which green HRM practices foster employees' environmental commitment by enhancing their sense of organizational pride and identification?*

CSR activities, in general, increase the sense of pleasure one feels in being a part of one's organization [16]. Being a combination of CSR and HRM, it is argued that green HRM practices show concern about environmental issues and, thus, inculcate in workers feelings of pride. Employees who feel proud of being associated with the company have a greater sense of identification with it [2]. When employees feel a connection with their organization, it results in employees exhibiting favorable attitudes toward it and its activities [17]. Such employees feel a sense of responsibility to assist the environmental initiatives of their organization. The organization. The functional role of organizational pride and organizational identification as serial mediators between green HRM and environmental commitment can be justified through the social identity theory [18,19], implying that people obtain feelings of identity and belonging from membership in social groups, including organizations [20]. When an organization adopts green HRM practices and communicates its commitment to environmental sustainability, employees may feel a sense of pride in their organization and a stronger identification with its values and goals. Such feelings, in turn, may lead to increased environmental commitment among employees. This study seeks to accomplish the following research objectives:

1. To investigate the mechanisms underlying the relationship between green HRM and environmental commitment.
2. To investigate the mediating role of organizational pride in the relationship between green HRM practices and environmental commitment.
3. To explore the mediating role of organizational identification in linking green HRM practices to environmental commitment.
4. To examine the sequential mediation of organizational pride and organizational identification in the association between green HRM practices and environmental commitment.

## 2 Literature review

### 2.1 Green HRM

One of the major business responses to the world's current environmental situation has been the introduction of green HRM [21]. Green HRM is defined as a collection of employee-focused strategies and practices aimed at developing and sustaining the workforce's motivation and skills, while providing them with opportunities to contribute to the organization's economic and environmental sustainability [22]. Empirical evidence has demonstrated its impact on employee workplace green behaviors and outcomes [7,15,[23], [24], [25]]. Human resource management exists to positively influence employee work outcomes, and this relationship occurs via various underlying processes [26]. Given the pressing nature of environmental concerns, there is significant research currently seeking to understand these underlying mechanisms better, in a manner that is advancing our understanding of green HRM beyond what is known about general HRM practices. Many potential mediating factors are being examined. For instance, considering the potential causal factor of leadership alone, we can see a significant amount of recent work looking into the mediating effects of visionary leadership [27], green transformational leadership [28] environmentally specific servant leadership [29], and leadership beliefs [15], among others. This illustrates the response to the continuing calls from the field for further exploration of the intervening mechanisms that link the implementation of green HRM to improved performance outcomes [15].

### 2.2 Employee environmental commitment (EEC)

As an antecedent of pro-environmental behaviors, environmental attitudes have long been an area of focus [30], and continued calls for more nuanced understandings of these antecedents persist [31,32]. In the words of Raineri and Paillé [33] environmental commitment is a mindset that indicates both a feeling of connection and obligation towards environmental issues within the workplace (p. 133). Employee commitment has long been a sought-after outcome of HRM practices [34] and has been shown to be a mediating variable between HRM systems and organizational outcomes [35,36]. However, the environmental dimension has remained under-examined [37]. Recently, Ren et al. [15] have argued and empirically supported the case that EEC specifically mediates “the relationship between green HRM and both the environmental performance and the financial performances of organizations” (p. 80). While there is growing interest in the mediating/antecedent role of EEC and employee performance and organizational outcomes, the mechanisms between green HRM and EEC require more profound elucidation. Our research seeks to examine some potentially high-value mediators between these two variables.

What can drive this discretionary commitment in employees, and what occurs between green HRM practices and the resulting levels of EEC? Raineri and Paillé [33] have illustrated that corporate environmental policy is more influential on EEC than individual ecological beliefs. Is that relationship mediated in any way? This study seeks to see if organizational pride and identification may provide further “black-box” answers.

### 2.3 Organizational pride (OP)

Organizational pride (OP) has been measured by the extent to which individuals feel a sense of satisfaction and self-esteem that comes from being a part of the organization [2]. We can distinguish organizational pride and organizational identification by pointing out that one can identify with one's organization without that positive feeling toward it [2,38]. De Roeck and Delobbe [39] explained the power of OP using social identity theory [19]. If employees believe that others value their company's practices (e.g., its environmental efforts), they feel more pride and exhibit greater citizenship behaviors.

Organizations that want to enhance positive employee behavior should pay attention to improving employees' pride [40]. A recent study [41] revealed organizational pride as a mediating mechanism between employees' CSR perception and organizational citizenship behavior. Organizational pride emphasizes the association between employees with their organization [42,43]. However, organizational pride remains one of the less explored areas in management and organizational behavior research [44,45], despite being considered a significant factor for business success [46] due to its impact on important job outcomes [47]. Social identity theory advocates that the employees' assessment of organizational CSR engagement has a positive impact on workers' organizational pride [16]. Organizational CSR activities significantly contribute to the company's reputation [48]. Employees feel proud in being part of a company that contributes to society and the environment because it adds to their self-worth and self-esteem [2]. Edwards and Shipp [49] also argued that individuals often prefer to be a part of organizations that implement CSR activities because it enhances their self-esteem. Therefore, it can be suggested that organizations prioritizing social responsibility tend to have higher levels of organizational pride among their employees, which, in turn, can influence pro-environmental behaviors and actions. We argued that employees who experience a sense of pride towards their workplace are more likely to adopt the values and goals of the organization, including those related to environmental sustainability.

• *Hypothesis H1. Organizational pride (OP) intervenes in the association between green HRM and EEC.*

### 2.4 Organizational identification (OI)

Organizational identification (OI) can be defined as a sense of unity or affiliation with an organization, where an individual defines themselves in relation to the organization(s) to which they belong [50]. Earlier conceptualizations saw it as a combination of the processes of self-categorization and self-enhancement [18,20], but these have come to be viewed separately as OP and OI [2,38]. Based on the findings of Ashforth and Mael [51], Jones [2] discussed that employees who experience a sense of pride towards their organizational membership are inspired to identify themselves with that organization to boost their self-esteem. Whereas, Organizational identification is described as feeling a sense of unity or connection with an organization and identifying oneself as a part of that organization [52].

Several authors have researched the antecedents of organizational identification, finding statistical significance in a variety of factors: prestige, support from superiors, opportunities for career advancement, access to the organizational hierarchy [53], perceived organizational support [54], perceived supervisor support, job satisfaction [55], job involvement [56] and the attractiveness of perceived organizational identity (APOI) [57]. Social identity theory posits that a positive perception of the working environment can boost employee identification with their companies [38]. According to Dutton et al. [38], an individual is closely associated with an organization when their identity as a member of the organization stands out more prominently than other identities. Kraemer and Gouthier [58] reasoned that organizational pride is directly linked to employee self-worth and self-esteem, which is valuable for an organization. Workers take pride in being affiliated with a company that enjoys a positive standing and image [51,59]. Schaefer et al. [16] indicate that employees are expected to feel pride in working for an organization involved in social responsibility activities, and as a result, these employees identify themselves with that organization.

Newman et al. [60] pointed out that organizations can increase employee identification by implementing HRM practices that encourage employee involvement in CSR activities aimed at benefiting various stakeholders, including the local community. The link between green HRM and Organizational identification can be justified through the support of social identity theory [38,51,61]. Green HRM is expected to contribute toward employees' self-esteem, leading to higher organizational identification [19]. Shen and Benson [61] argued that employees assess their organization based on its engagement in CSR activities; if employees perceive it positively, it fosters an increased level of organizational identification. Employees exposed to environmentally friendly policies and practices in the workplace may feel a greater sense of pride and commitment to the organization, seeing it as a responsible and ethical place to work. Furthermore, workers who have the opportunity to participate in the company's sustainability initiatives may experience a deeper sense of possession and connection to the company.

• *Hypothesis H2. Organizational identification (OI) intervenes to connect green HRM with EEC.*

### 2.5 Role of organizational pride and organizational identification as sequential mediators between green HRM and employees’ environmental commitment

**Fig. 1:**
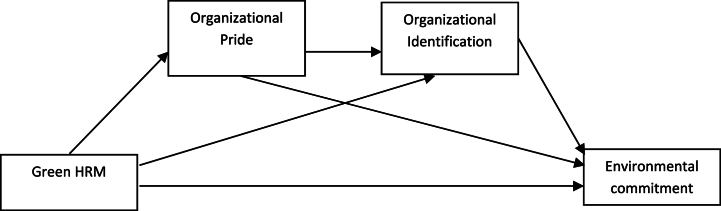
Theoretical framework.

Reade [53] noted the wide range of potential antecedent factors and how different factors may be significant in different contexts. Complex mediation models are now being seen in the literature [62]. Tüzün and Çağlar [57] showed the attractiveness of perceived organizational identity and identification to be mediated by trust. The complexity of the context and the potential for sequential mediation have prompted our research to examine whether OP and OI could serve as intermediate factors in the link between green HRM and EEC. Researchers [63,64] have also identified OP to be a factor in the sequential mediation mechanism between employees’ perception of corporate social responsibility and organizational citizenship behaviors. Revealing any sequential mechanisms through which green HRM affects employee environmental commitment can provide valuable insights into refining green HRM practices for more effective targeting. The theoretical framework is presented in Fig. 1.

We argue that implementing Green HRM leads individuals to feel proud of their organization for taking steps to be more sustainable and socially responsible. This increased pride in the organization may lead individuals to feel more strongly identified with the organization and its values. This increased organizational identification may, in turn, lead individuals to feel more committed to upholding the organization's commitment to sustainability and social responsibility through their own behavior and actions.

Based on the above-referenced literature, the following hypotheses are proposed:

• *Hypothesis H3. OP and OI intervene sequentially in the connection between green HRM and EEC.*

## 3 Method

### 3.1 Participants and procedure

The population of this study comprised non-managerial employees working for telecom sector companies in Pakistan. The telecom sector was chosen for its active participation in CSR-related activities. The company's commitment to CSR was verified through an examination of its websites and annual reports. Additionally, our assessment is consistent with the study of Javed and Khan [65], affirming the active involvement of Pakistan's major cellular service companies in CSR initiatives. The reason for considering only non-managerial employees for this study is that they are unlikely to participate in the developmental stage of CSR. Data collection took place from June 2020 through July 2020. The data were collected through personally administered questionnaires in Punjab, the most populated province in Pakistan. A total of 523 surveys were handed out to non-managerial staff. The surveys collected were anonymous, and all participants provided written informed consent. The survey was voluntary for the participants, and they were guaranteed that their answers would be kept private and used only for academic purposes to address the issue of social desirability [66]. Most of the questionnaires were distributed and collected back by hand, with only a limited number sent through a courier service. The survey was carried out in English since it's considered an official language in Pakistan, and higher education in the country is conducted in English. Therefore, most educated people find English easy to read and understand. Out of the 523 questionnaires distributed, 310 were returned with a response rate of 59 % and a total of 267 were finally accepted for data analysis. 5-point Likert-type scales were employed to collect the data against each item, where 1 represents ‘strongly disagree’ and 5 represents ‘strongly agree’. Table 1 provides an overview of the participant's demographic characteristics.

**Table 1:** Demographic characteristics of respondents.

Characteristics	Frequency	Percentages (%)
**Gender**		
Male	211	79
Female	56	21
**Age (Years)**		
18-28	99	37.1
29-39	160	59.9
40 and above	8	3.0
**Education**		
High school	8	3.0
Bachelor's degree	214	80.1
Master and above	45	16.9
**Tenure**		
1-3	88	33
4-6	40	15
7-9	49	18.4
Over 10 years	90	33.7

The data were analyzed using PLS-SEM in smart PLS 3.0. The reasons to use PLS-SEM are its suitability with a small sample size and its handling of non-normal data [67]. Although PLS-SEM is usually recommended for exploratory studies, it can also be applied in confirmatory research [68].

### 3.2 Measures

The green HRM questionnaire consists of six items adopted from the study of Dumont et al. [24]. Examples of items are “My company sets green goals for its employees” and “My company provides employees with green training to develop employees' knowledge and skills required for green management.” The organizational pride questionnaire consists of four items from Jones [2]. Examples of items are “I am proud to work for my organization” and “I am proud of what my organization accomplishes.” The measure of organizational identification consists of six items from the work of Mael and Ashforth [52]. Included among these items are statements such as “When someone praises my organization, it feels like a personal compliment” and “I usually say ‘we’ rather than ‘they’.” The measure of environmental commitment consists of eighteen items adopted from Raineri and Paillé [33]. Examples of items are “The environmental concern of my organization means a lot to me” and “I feel an obligation to support the environmental efforts of my organization.”

### 3.3 Analysis and results

#### 3.3.1 Harman's single-factor test

When data is collected from a single source, it may lead to the issue of common method variance (CMV) [69]. In this study, data were collected solely from non-managerial employees of telecom sector companies. To assess the presence of CMV, Harman's single-factor analysis was employed. If a significant proportion of variance among the variables can be attributed to a single factor, it indicates a potential CMV issue [69]. The results of Harman's single-factor analysis show that only 32.91 % of the variance can be explained by one fixed factor, which is below the maximum threshold of 50 %. Therefore, based on this result, we can conclude that there is no issue of common method variance in this data.

#### 3.3.2 The measurement model

**Fig. 2:**
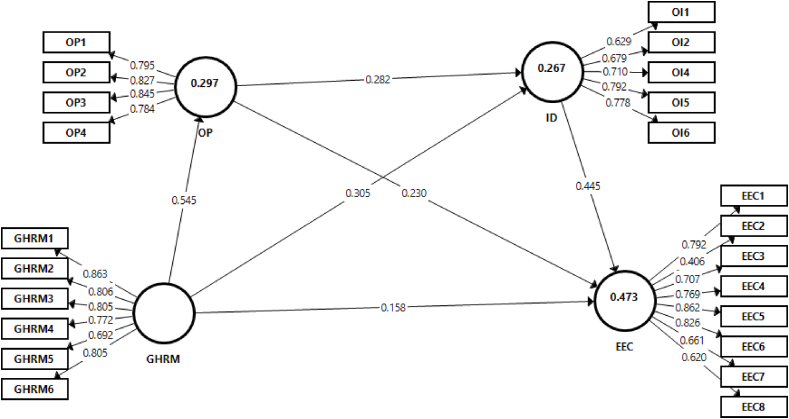
Estimation of the sequential mediation model.

The estimation of sequential mediation is given in Fig. 2. The initial phase involved assessing a measurement model, employing approaches such as convergent validity, discriminant validity, and internal consistency reliability as suggested by Hair et al. [70]. The reliability of the variables was assessed through the use of composite reliability and Cronbach alpha values. As Table 2 demonstrates, the Cronbach alpha values range from 0.784 to 0.896, and composite reliability values range from 0.843 to 0.910. All values exceed the threshold of 0.70, indicating satisfactory reliability [71].

**Table 2:** Description, Factor loadings, reliability, and AVE.

Variables	Items	Mean	SD	Load-ings	Cron-bach’ s Alpha	rho_A	CR	AVE
Green HRM (GHRM)	GHRM1	3.914	0.876	0.863	0.882	0.898	0.910	0.627
	GHRM2	3.854	0.748	0.805				
	GHRM3	3.891	0.734	0.804				
	GHRM4	3.742	0.747	0.772				
	GHRM5	3.727	0.805	0.693				
	GHRM6	3.584	0.905	0.805				
Employee Environmental Commitment (EEC)	EEC1	3.869	0.831	0.791	0.872	0.891	0.901	0.568
	EEC3	3.88	0.719	0.703				
	EEC4	3.854	0.885	0.767				
	EEC5	3.704	0.797	0.860				
	EEC6	3.775	0.785	0.836				
	EEC7	3.693	0.867	0.664				
	EEC8	3.918	0.779	0.625				
Organizational Identification (OI)	OI1	3.67	0.946	0.628	0.769	0.784	0.842	0.519
	OI2	3.637	0.856	0.678				
	OI4	4.142	0.892	0.712				
	OI5	3.854	0.955	0.792				
	OI6	3.697	0.858	0.778				
Organizational Pride (OP)	OP1	4.288	0.736	0.795	0.829	0.830	0.886	0.661
	OP2	4.049	0.831	0.827				
	OP3	4.131	0.746	0.845				
	OP4	4.075	0.785	0.783				

N = 267 AVE = Average variance extracted, CR=Composite reliability.

Convergent validity was examined using outer loadings of all the items of observed variables and the average variance extracted (AVE) of all variables. The results revealed that the factor loadings of all indicators were higher than the cut-off limit of 0.50 [72]. Two items with low outer loadings had been deleted. In the next step, convergent validity was evaluated using AVE values. The AVE values of all variables ranged from 0.519 to 0.661, surpassing the 0.50 cutoff [73]. Thus, there are no issues of convergent validity.

Discriminant validity was evaluated by using the heterotrait-monotrait ratio (HTMT ratio) and Fornell-Larker criterion as stated by Henseler et al. [74]. The Fornell-Larker criterion results are presented in Table 3, which indicates that the correlation between each variable and any other variable is lower than the square root of its average variance extracted (AVE) [75]. The HTMT ratio is a contemporary method to evaluate discriminant validity in comparison to the Fornell-Larker criterion [76]. The HTMT ratio, a contemporary method for evaluating discriminant validity, is provided in Table 4, where all values are below the maximum cutoff of 0.90. Therefore, discriminant validity has been successfully established.

**Table 3:** Discriminant validity based on the Fornell-Larker criterion.

Variables	EEC	GHRM	ID	OP
Employee environmental commitment (EEC)	**0.754**			
Green HRM (GHRM)	0.498	**0.792**		
Organizational identification (OI)	0.619	0.459	**0.720**	
Organizational pride (OP)	0.525	0.545	0.449	**0.813**

* The diagonal of the data displays the square roots of the AVE, while the off-diagonal values represent the correlations between the constructs.

**Table 4:** Discriminant validity based on the Heterotrait-monotrait ratio.

Variables	EEC	GHRM	ID	OP
Employee Environmental commitment (EEC)				
Green HRM (GHRM)	0.532			
Organizational identification (OI)	0.736	0.514		
Organizational pride (OP)	0.591	0.621	0.535	

Additionally, Table 5 reveals that the variance inflation factors (VIF) are all below the threshold of 5, indicating no concerns of potential multicollinearity among the study's variables. Therefore, we can confidently proceed with our further analysis.

**Table 5:** Multicollinearity statistics (VIF).

	EEC	GHRM	ID	OP
EEC				
GHRM	1.550		1.423	1.000
ID	1.364			
OP	1.531		1.423	

#### 3.3.3 Hypotheses testing

After fulfilling the requirements of the measurement model, the structural model was run to examine the hypotheses using the bootstrap method incorporating 5000 resamples. The coefficient of determination (R^2^) for the main dependent variable (environmental commitment) was used to assess the model's predictive power. The R^2^ value for environmental commitment is 0.478, which is considered as moderate based on the criteria suggest by Hair et al. [71].

The results regarding direct and indirect relationships are given in Table 6. The results indicate that green HRM is positively related to organizational pride (β = 0.545, p < 0.05) *LLCI* = 0.436, *ULCI* = .638. The results further indicate that the direct effect of green HRM on organizational identification is significant (β = 0.31, p < 0.05) *LLCI* = 0.191, *ULCI* = .416. The impact of organizational pride on identification is also significant (β = 0.29, p < 0.05) *LLCI* = 0.179, *ULCI* = .392. Moreover, the link between organizational pride and environmental commitment is significant (β = 0.23, p < 0.05) *LLCI* = 0.140, *ULCI* = .325. Likewise, the effect of organizational identification on environmental commitment (β = 0.45, p < 0.05) *LLCI* = 0.329, *ULCI* = .556 is also significant.

**Table 6:** Hypothesis testing results summary.

	*B*	*T*		*p*		*LLCI*		*ULCI*
**GHRM -** > **OP**	0.545	10.554		0.000		0.436		0.638
**GHRM -** > **ID**	0.308	5.278		0.000		0.191		0.416
**OP -** > **ID**	0.287	5.205		0.000		0.179		0.392
**OP -** > **EEC**	0.233	4.849		0.000		0.140		0.325
**ID -** > **EEC**	0.447	7.781		0.000		0.329		0.556
*The indirect effects of GHRM on environmental commitment*								
	***B***	***T***	***p***		***LLCI***		***ULCI***	
**Indirect 1: GHRM -** > **OP -** > **EEC**	0.127	4.176	0.000		0.073		0.190	
**Indirect 2: GHRM -** > **OI -** > **EEC**	0.138	4.025	0.000		0.074		0.209	
**Indirect 3: GHRM -** > **OP -** > **OI-** > **EEC**	0.070	4.197	0.000		0.040		0.103	

The first hypothesis proposed that the impact of green HRM on environmental commitment would be mediated by organizational pride. The results indicate that the effect of green HRM on environmental commitment through organizational pride is significant (β = 0.13, p < 0.05) *LLCI* = 0.073, *ULCI* = .190, supporting H1. Further, the results of hypothesis 2 support the indirect influence of green HRM on environmental commitment through organizational identification (β = 0.14, p < 0.05) *LLCI* = 0.074, *ULCI* = .209. The final hypothesis proposed that the sequential mediation of organizational pride and organizational identification would serve as mediating factors in the indirect relationship between green HRM and environmental commitment. The analysis reveals that this relationship is also significant (β = 0.07, p < 0.05), with LLCI = 0.040 and ULCI = 0.103. Thus, organizational pride and identification sequentially mediated the impact of green HRM on environmental commitment. Hence, hypothesis H3 is also supported.

## 4 Discussion

Pro-environmental behaviors are key aspects of modern business. The current environment is experiencing a significant decline, which has prompted governments and organizations to put into place effective measures to address this issue [77]. Following on from such legitimate, proximal environmental concerns, “corporate greening” [33] has become a central business driver in the last few decades. Irrespective of whether environmental concerns are genuinely central to a company's core ethos, wider public opinion directs businesses to respond to such concerns [78], with many organizations strongly pushing to become more environmentally sustainable [79]. Such responses have become a key business strategy [80], leading to increased sales, improved brand recognition, and achieving favorable results for employees [24,81,82] and the achievement of sustainability goals [83].

This research was conducted to explore the complicated psychological mechanisms underlying the link between green HRM and employee environmental commitment. Specifically, the present study has examined a sequential mediation model connecting green HRM and environmental commitment through the mediating variables of organizational pride and identification based on social identity theory. This study is pioneering in exploring the connection between green HRM and environmental commitment through organizational pride and identification routes in telecom companies in Pakistan.

In line with previous studies, the results supported the assumption that green 10.13039/100005448HRM positively affects an employee's level of environmental commitment [15]. In line with social identity theory [18,51], this study suggests that when employees notice that their organization's HR practices are environmentally friendly and socially responsible, they react by being proud to be part of that organization and are motivated to identify themselves with their organization to increase their sense of self-esteem [2,51]. This ultimately leads to increases in employee environmental commitment. The results of this study suggest a positive correlation between green human resource management (HRM) and a sense of organizational pride. Furthermore, there exists a positive association between green HRM and organizational identification. This result is in line with previous studies [60,61,84]. Further, there is a positive relationship between organizational pride and organizational identification, which is consistent with the results of Jones [2]. The results of the present study also reveal a positive relationship between organizational pride and environmental commitment. Moreover, the results demonstrate a positive link between organizational identification and environmental commitment, which is consistent with the study of Knight and Haslam [85]. Furthermore, by describing the sequential mediation effect of organizational pride and identification, where green HRM first fosters organizational pride, which, in turn, heightens the organizational identification of employees and, finally, results in an increase in employees' environmental commitment, this study contributes to the findings of Ren et al. [15], who investigated the direct effect of green HRM on environmental commitment. This sequential mediation effect is a clear application of the principles of social identity theory, demonstrating how the theory is reflected in the research findings. By positioning our results within the framework of social identity theory, we enhance the overall comprehension of the psychological mechanisms that green HRM enhances environmental commitment among employees in the telecommunications sector of Pakistan.

### 4.1 Implications

This research has implications both from a theoretical and practical perspective.

#### 4.1.1 Theoretical implications

As discussed earlier, the existing literature primarily focuses on exploring the direct relationship between green HRM and environmental commitment [15,16] but there is a gap in the literature regarding the underlying psychological mechanisms. This study carries important theoretical implications, suggesting the necessity to identify more potential mediators that can effectively drive behavioral changes. By identifying mediators that have stronger influences on behavioral changes [86], researchers have the opportunity to tap into more powerful mechanisms for encouraging desired behaviors. This requires further examination of already considered variables and the examination of as-yet neglected or potential variables or pathways of mediation (e.g., sequential mediation), allowing researchers to move toward the development of an understanding of the causal process for behavioral change [87,88]. This study contributes to understanding the effect of green HRM on employee environmental commitment through an examination of the mediating roles of organizational pride and organizational identification sequentially.

In the context of social identity theory, this study enhances our understanding by examining the sequential mediating roles of organizational pride and organizational identification. These findings suggest that employees' sense of pride in their organization and their identification with it can be powerful drivers of environmental commitment when coupled with green HRM practices. This not only validates the social identity theory but also supports its practical application in the field of HRM. Finally, by focusing on Pakistan's telecom sector, this study addresses a contextual gap in the literature. It offers a deep understanding of how employees in this specific context perceive green HRM and how the sequential mediation of organizational pride and identification influences the impact of green HRM on environmental commitment. This context-specific insight could potentially guide the adaptation and application of social identity theory in similar cultural or industrial contexts.

#### 4.1.2 Managerial implications

Although the data for this study were collected from telecom sector companies in Pakistan, it still has implications for managers and practitioners in general because consumers and stakeholders are increasingly demanding that organizations prioritize sustainability. Organizations can strengthen their relationships with key stakeholders and improve their reputation by demonstrating their commitment to environmental responsibility, through the adoption of green HRM practices. Overall, this research exploring the serial mediation of organizational pride and organizational identification between green HRM and environmental commitment, can provide scholars with a deep understanding of the core mechanisms through which green HRM practices influence environmental commitment. It can also help organizations to better understand the conditions under which green HRM practices are most effective. Identifying the potential mediators may also benefit organizations and the environment, as these mediators can be more successfully manipulated through interventions, practices, or policies that improve employee-driven environmental outcomes. Companies can enhance their environmental performance by adopting green HRM practices, which foster increased environmental commitment among employees. These environmentally friendly initiatives significantly add to the company's environmental performance [7]. This is also important for companies that are aiming to achieve their sustainability objectives and minimize their environmental impact. This is probably because employees, who perceive their organization to be environmentally responsible, tend to demonstrate greater organizational pride and identification.

### 4.2 Limitations and future research directions

This research has certain shortcomings that future studies could address. Firstly, this study used a cross-sectional approach to collect data, so the full impact of green HRM on employee environmental commitment has yet to be fully explored. Future studies could employ a longitudinal research design to analyze changes in employee environmental commitment resulting from the implementation of green HRM. Secondly, the research took place in Pakistan, which has a unique culture and is classified as a developing country. Potential future studies could examine the same model in developed economies or different cultures to confirm the findings of this study. Research has demonstrated that a country's culture may significantly affect the direction and intensity of the linkage between variables [89,90]. Further, future studies could investigate the other mechanisms between green HRM and employee environmental commitment i.e., organizational trust [91]. Finally, the model of this study could be extended to include additional green outcomes for employees, such as green creativity, green knowledge sharing, and environmental performance.

### 4.3 Conclusion

In a world facing the dual challenges of diminishing natural resources and environmental degradation, CSR has emerged as an essential strategy for providing competitive advantage specifically in building brand reputation and managing talent. Green HRM and CSR are linked concepts that can be used by a company to assess the effects of its operation on the environment and society. Although the literature well documents the strong relationship between green HRM and environmental commitment, it is unclear how green HRM influences employee environmental commitment. Specifically, it is unclear how organizational pride, and organizational identification influence this relationship. The key findings of this study indicate that green HRM practices can significantly boost employees’ environmental commitment, with the strengthening of organizational pride and identification plays a crucial role in this process. This finding directly addresses our research question by demonstrating the importance of organizational pride and identification in promoting environmental commitment. In view of these findings, we propose that organizations should prioritize environmental initiatives and communicate their efforts to workers. This strategy will be helpful in cultivating a sense of organizational pride and identification, which can, in turn, increase employee environmental commitment. By doing so, organizations can promote a culture of responsibility towards green initiatives, thereby contributing to the broader goal of environmental sustainability.

## Funding statement

This work was supported and funded by the Deanship of Scientific Research at Imam Mohammad Ibn Saud Islamic University (IMSIU) (grant number IMSIU-RG23053).

## Data availability statement

Data will be made available on request.

## CRediT authorship contribution statement

**Saeed Turki Alshahrani:** Writing – review & editing, Writing – original draft. **Kamran Iqbal:** Formal analysis, Conceptualization.

## Declaration of competing interest

The authors declare that they have no known competing financial interests or personal relationships that could have appeared to influence the work reported in this paper.
